# Dr. William Oliver

**Published:** 1902-08

**Authors:** 


Dr. William Oliver, of Penn Yan, a graduate of Geneva Medical College in 1846, died suddenly from an affection of the heart July 10, 1902, aged 79 years. He had practised his profession for more than half a century in the village where he died.

